# Postoperative Complications After Neoadjuvant Chemotherapy Versus Upfront Surgery in Gastric Adenocarcinoma: A Population-Based Nationwide Study in Finland

**DOI:** 10.1245/s10434-023-14813-5

**Published:** 2023-12-28

**Authors:** Emilia Putila, Olli Helminen, Mika Helmiö, Heikki Huhta, Aapo Jalkanen, Raija Kallio, Vesa Koivukangas, Arto Kokkola, Simo Laine, Elina Lietzen, Johanna Louhimo, Sanna Meriläinen, Vesa-Matti Pohjanen, Tuomo Rantanen, Anna Junttila, Ari Ristimäki, Jari V. Räsänen, Juha Saarnio, Eero Sihvo, Vesa Toikkanen, Tuula Tyrväinen, Antti Valtola, Joonas H. Kauppila, the FINEGO Group

**Affiliations:** 1 Surgery Research Unit, Department of Surgery, Medical Research Center Oulu, University Hospital and University of Oulu, Oulu, Finland; 2 Division of Digestive Surgery and Urology, Turku University Hospital, Turku, Finland; 3 Department of Surgery, University of Helsinki and Helsinki University Hospital, Helsinki, Finland; 4 Department of Oncology and Radiotherapy, Oulu University Hospital, Oulu, Finland; 5 Cancer and Translational Medicine Research Unit, Medical Research Center Oulu, University of Oulu and Oulu University Hospital, Oulu, Finland; 6 Department of Surgery, University of Eastern Finland and Kuopio University Hospital, Kuopio, Finland; 7 Department of Surgery, Kuopio University Hospital, Kuopio, Finland; 8 Department of Pathology, HUSLAB, HUS Diagnostic Center, Helsinki University Hospital and University of Helsinki, Helsinki, Finland; 9 Applied Tumor Genomics Research Program, Research Programs Unit, Faculty of Medicine, University of Helsinki, Helsinki, Finland; 10 Department of General Thoracic and Oesophageal Surgery, Heart and Lung Centre, University of Helsinki and Helsinki University Hospital, Helsinki, Finland; 11 Department of Surgery, Central Finland Central Hospital, Jyväskylä, Finland; 12 Department of Cardiothoracic Surgery, Heart Center, Tampere University Hospital and University of Tampere, Tampere, Finland; 13 Department of Gastroenterology and Alimentary Tract Surgery, Tampere University Hospital, Tampere, Finland; 14 Department of Molecular Medicine and Surgery, Karolinska Institutet and Karolinska University Stockholm, Stockholm, Sweden

## Abstract

**Background:**

To date, no large population-based studies have compared complications and short-term outcomes between neoadjuvant chemotherapy and upfront surgery in gastric cancer. More nationwide studies with standardized reporting on complications are needed to enable international comparison between studies. This study aimed to compare postoperative complications between neoadjuvant therapy and upfront surgery after gastrectomy for gastric adenocarcinoma in a population-based setting.

**Methods:**

This population-based study based on the Finnish National Esophago-Gastric Cancer Cohort included all patients 18 years of age or older undergoing gastrectomy for gastric adenocarcinoma in Finland during 2005–2016. Logistic regression provided odds ratios (ORs) with 95% confidence intervals (CIs), both crude and adjusted for key confounders. Different types of complications were graded based on the Esophagectomy Complications Consensus Group definitions, and major complications were assessed by the Clavien-Dindo scale.

**Results:**

This study analyzed 769 patients. Neoadjuvant chemotherapy did not increase major postoperative complications after gastrectomy for gastric cancer compared with upfront surgery (OR, 1.12; 95% CI 0.81–1.56). Furthermore, it did not increase pneumonia, anastomotic complications, wound complications, or other complications.

**Conclusions:**

Neoadjuvant therapy is not associated with increased postoperative complications, reoperations, or short-term mortality compared with upfront surgery in gastric adenocarcinoma.

Gastric cancer is the third leading cause of cancer death worldwide, with up to 800,000 annual deaths.^1,2^ Because early gastric cancer is often asymptomatic, the majority of patients have an advanced stage of disease at diagnosis.^3^ The cornerstone of gastric adenocarcinoma treatment is multimodality management including gastrectomy with lymphadenectomy accompanied by preoperative cytotoxic therapy.^4^ Gastrectomy is associated with frequent complications and high mortality rates.^5^

Neoadjuvant chemotherapy for gastric adenocarcinoma downstages the tumor and improves both progression-free and overall survival.^6,7^ The European Society for Medical Oncology (ESMO) guidelines recommend perioperative chemotherapy for patients with stage ≥ IB resectable gastric cancer.^4^ It is, however, unknown whether surgical risks in neoadjuvant-treated patients are increased outside the selected clinical trial populations.

Only a few small studies have investigated postoperative complications after gastrectomy for gastric cancer comparing neoadjuvant chemotherapy with upfront surgery. A Chinese retrospective study (*n* = 170) suggested no significant difference in postoperative complications in a comparison of neoadjuvant chemotherapy (18.8%) with upfront surgery (22.2%).^8^ Another Chinese retrospective study (*n* = 377) suggested fewer postoperative complications for patients receiving neoadjuvant treatment (10.0%) instead of upfront surgery (17.2%). The most common complications are motility and pulmonary problems, intra-abdominal abscess, and anastomotic leak.^9^ However, no nationwide studies, large European studies, or studies using standardized definitions of complications exist on this topic.

This study aimed to compare postoperative complication rates after gastric cancer resection for patients receiving neoadjuvant therapy compared with upfront surgery in a population-based setting.

## Methods

### Study Design

This study was a population-based retrospective cohort study in Finland using the Finnish National Esophago-Gastric Cancer Cohort (FINEGO).^10^ For the current analysis, the study enrolled patients who underwent curatively intended gastrectomy for clinical stage ≥ IB gastric adenocarcinoma during 2005–2016.

**Fig. 1:**
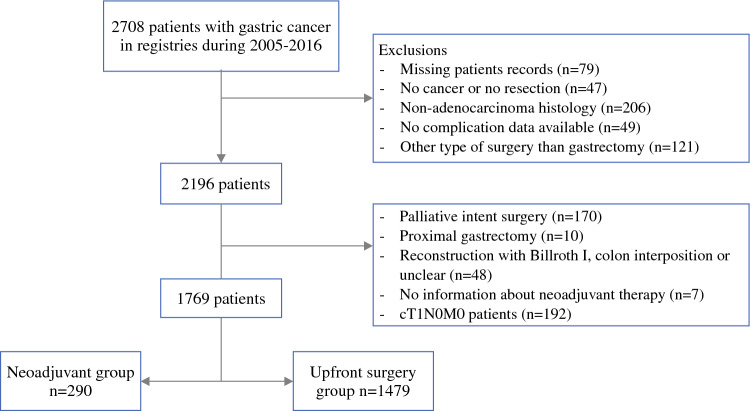
Patient selection criteria

The inclusion criteria for the patients specified a diagnosis of gastric cancer, surgical treatment for the diagnosed adenocarcinoma, and age 18 years or older at the time of diagnosis. The exclusion criteria ruled out patients with proximal gastrectomy, Billroth I reconstruction, colonic interposition, or early gastric cancer (clinical stage IA), as well as patients with missing data on complications or neoadjuvant treatment. Patient selection and exclusion criteria are presented in Fig. 1.

### Data Collection

The reliable and complete Finnish Cancer Registry and Finnish Patient Registry were used to identify all potentially eligible patients.^11,12^ Patient records for patients with gastric cancer or tumor diagnosis in the Finnish Patient registry or the Finnish Cancer Registry and a relevant surgical code in the Patient Registry were retrieved from the respective health care units and hospitals and screened for eligibility by expert surgeons.^13^

Data on age, sex, date of surgery and diagnoses were provided by the Patient Registry. Charlson comorbidity was calculated based on diagnoses in the patient registry using the validated and the most up-to-date version of the Charlson Comorbidity Index.^14^ Expert upper gastrointestinal surgeons evaluated patient records, including surgical charts and pathology assessments. Cancer stage information was updated according to tumor-node-metastasis (TNM) 8.^15^ Information on tumor and treatment characteristics and complications was retrieved and inputted to the common database using Research Electronic Data Capture (REDCap), a web-based tool hosted at the University of Oulu, Finland.^16,17^ Statistics Finland provided the 100% complete and reliable mortality data.^18^

### Exposure

The exposure was neoadjuvant therapy compared with upfront surgery (reference). The neoadjuvant therapy for 94.1% of the patients comprised EOX-derived triple therapies (including EOF, ECX, and ECF). Five patients received XELOX, whereas three patients received XELOX accompanied by Herceptin, and nine patients received other regimens.

### Outcomes

The primary outcome was the occurrence of major complications, defined as Clavien-Dindo grade IIIa or higher.^19^ The secondary outcomes were pneumonia, anastomotic leak, wound dehiscence, and complications grouped by the Esophagectomy Complications Consensus Group (ECCG) (pulmonary, cardiac, gastrointestinal, thromboembolic, neurologic, urologic, infectious, wound, or other), and 90-day reoperations. Finally, 90-day mortality was examined to evaluate competing risks due to mortality.

The severity of complications was classified according to the Clavien-Dindo classification. The ECCG classification of postoperative complications was used to single out each complication type and to classify the complications in upper-level complication categories (pulmonary, cardiac, gastrointestinal, thromboembolic, neurologic, urologic, infectious, wound, or other). For different types of complications after gastric cancer surgery, previous nationwide analyses have been reported using the ECCG standardized list of complications.^20^ Reoperations were defined as surgical interventions in the operation theater performed with or without general anesthesia.

### Statistical Methods

Statistical analysis was performed according to a detailed prior study protocol. Patient characteristics, total and individual complications, and lengths of the postoperative intensive care unit (ICU) and hospital stays are presented stratified by neoadjuvant therapy. For the *p* values of patient characteristics, group variables were compared using the chi-square test, and continuous variables were compared using the Mann-Whitney *U* test. Logistic regression provided odds ratios (ORs) with 95% confidence intervals (CIs), both crude and adjusted for the confounders. Confounders were age (continuous), sex (male or female), Charlson Comorbidity Index (CCI 0, 1, 2, or 3 ≥), year of surgery (continuous), pathologic cancer stage (0, I, II, III, or IV), surgical technique (open or laparoscopic), and type of gastrectomy (total or distal).

For survival outcomes, Cox regression provided hazard ratios (HRs) with 95% CIs, both crude and adjusted for confounders. To account for the nutritional status of the patients, an additional analysis adjusted for body mass index (BMI) (abnormal or normal), and albumin or prealbumin (abnormal or normal) was determined in addition to the aforementioned confounders. Sensitivity analyses with adjustment for clinical instead of pathologic stage as well as for patients with R0 resection only were performed. To account for missing data, multiple imputation was performed for confounding variables with 20 iterations, assuming that the values were missing at random. Because complete case analysis did not differ from analyses with multiple imputation, only the analyses with multiple imputation are presented.

## Results

### Patients

From the registries, the study identified 2708 patients who had gastric cancer during 2005–2016. After exclusions (Fig. 1), the study enrolled 1769 patients undergoing gastrectomy for clinical stage IB or greater gastric adenocarcinoma. Of these 1769 patients, 290 (16.4%) received preoperative neoadjuvant treatment and 1479 (83.6%) underwent upfront surgery.

Patient characteristics are described in Table 1. The median age of all the patients was 70 years. Those who had neoadjuvant treatment were younger and had fewer comorbidities, lower pathologic stage disease, and more total gastrectomy and D2 lymphadenectomy than the patients in the upfront-surgery group. Duration of surgery and bleeding during surgery were similar between the groups.

**Table 1:** Characteristics of the 1769 patients undergoing gastrectomy for gastric cancer

	Neoadjuvant therapy			*p* value
	Yes	No	Whole cohort	
	(*n* = 290)	(*n* = 1479)	(*n* = 1769)	
	*n* (%)	*n* (%)	*n* (%)	
Year of surgery				**< 0.001**
Median (IQR)	2013 (2011–2015)	2009 (2007–2012)	2010 (2007–2013)	
Age (years)				**< 0.001**
Median (IQR)	65 (59.5–70.5)	71 (63–79)	70 (62–78)	
Sex				0.814
Male	159 (54.8)	822 (55.6)	981 (55.5)	
Female	131 (45.2)	657 (44.4)	788 (44.5)	
CCI				**0.003**
0	157 (54.1)	767 (51.7)	921 (52.1)	
1	101 (34.8)	427 (28.9)	528 (29.8)	
2	23 (7.9)	170 (11.5)	193 (10.9)	
≥ 3	9 (3.1)	118 (8.0)	127 (7.2)	
Pathologic (yp/pTNM) stage				**< 0.001**
0–I	100 (34.5)	304 (20.6)	404 (22.8)	
II	78 (26.9)	458 (31.0)	536 (30.3)	
III	82 (28.3)	572 (38.7)	654 (37.0)	
IV	26 (9.0)	117 (7.9)	143 (8.1)	
Missing	4 (1.4)	28 (1.9)	32 (1.8)	
Surgical technique				0.739
Open	277 (95.5)	1419 (95.9)	1696 (95.9)	
Laparoscopic	13 (4.5)	60 (4.1)	73 (4.1)	
Type of gastrectomy				**< 0.001**
Total	224 (77.2)	927 (62.7)	1151 (65.1)	
Distal	66 (22.8)	552 (37.3)	618 (34.9)	
Type of lymphadenectomy				**< 0.001**
D0	11 (3.8)	165 (11.2)	176 (9.9)	
D1	95 (32.8)	739 (50.0)	834 (47.1)	
D2	179 (61.7)	532 (36.0)	711 (40.2)	
Missing	5 (1.7)	43 (2.9)	48 (2.7)	
BMI				0.889
Normal	217 (74.8)	1164 (78.7)	1381 (78.1)	
Abnormal	18 (6.2)	93 (6.3)	111 (6.3)	
Missing	55 (19.0)	222 (15.0)	277 (15.7)	
Prealbumin				0.253
Normal	123 (42.4)	472 (31.9)	595 (33.6)	
Abnormal	47 (16.2)	224 (15.1)	271 (15.3)	
Missing	120 (41.4)	783 (52.9)	903 (51.0)	

*IQR* interquartile range, *CCI* Charlson Comorbidity Index, *TNM* tumor-node-metastasis, *BMI* body mass index

Statistically significant differences are in **bold** type

### Occurrence of Complications

The 90-day complication rate was 42.1%, and it was similar between the neoadjuvant patients (40.7%) and the upfront-surgery patients (42.4%). Major complications (Clavien-Dindo ≥ III) were similar between the two groups (17.9% of the patients in the neoadjuvant group and 16.3% of the patients in the upfront-surgery group; Table 2).

**Table 2:** Occurrence of complications for the 1769 patients undergoing gastrectomy for gastric cancer

	Neoadjuvant therapy		
	Yes	No	Whole cohort
	(*n* = 290)	(*n* = 1479)	(*n* = 1769)
	*n* (%)	*n* (%)	*n* (%)
90-Day complications	118 (40.7)	627 (42.4)	745 (42.1)
Major complications	52 (17.9)	241 (16.3)	293 (16.6)
Clavien-Dindo			
No complications or grade 1	173 (59.7)	853 (57.7)	1026 (58.0)
Grade 2	65 (22.4)	385 (26.0)	450 (25.4)
Grade 3	39 (13.4)	137 (9.3)	176 (9.9)
Grade 4	7 (2.4)	65 (4.4)	72 (4.1)
Grade 5^a^	6 (2.1)	39 (2.6)	45 (2.5)
ECCG 90-day complications			
Pulmonary	51 (17.6)	211 (14.3)	262 (14.8)
Pneumonia	34 (11.7)	167 (11.3)	201 (11.4)
Cardiac	**10 (3.4)**	**107 (7.2)**	117 (6.6)
Gastrointestinal	44 (15.2)	290 (19.6)	334 (18.9)
Anastomotic complication	10 (3.4)	69 (4.7)	79 (4.5)
Urologic	10 (3.4)	67 (4.5)	77 (4.4)
Thromboembolic	6 (2.1)	30 (2.0)	36 (2.0)
Neurologic	5 (1.7)	33 (2.2)	38 (2.1)
Infectious	**59 (20.3)**	**247 (16.7)**	306 (17.3)
Wound	6 (2.1)	29 (2.0)	35 (2.0)
Wound dehiscence	6 (2.1)	26 (1.8)	32 (1.8)
Other	8 (2.8)	29 (2.0)	37 (2.1)
Bleeding during surgery: ml (IQR)	400 (200–663)	400 (200–600)	400 (200–643)
Missing (%)	22 (7.5)	133 (9.0)	145 (8.2)
Surgery duration: min (IQR)	195 (150–239)	170 (130–213)	174.5 (134–217)
Missing (%)	12 (4.1)	117 (7.9)	129 (7.3)
Hospital stay: days (IQR)	9 (7–11)	9 (6.5–11.5)	9 (7–11)
Missing (%)	5 (1.7)	21 (1.4)	26 (1.5)
ICU stay: days (IQR)	0 (0–0)	0 (0–0)	0 (0–0)
Missing (%)	46 (15.9)	191 (12.9)	237 (13.4)
30-Day mortality	2 (0.7)	49 (3.3)	51 (2.9)
90-Day mortality	**10 (3.4)**	**95 (6.4)**	105 (5.9)

^a^In-hospital mortality

*ECCG* Esophagectomy Complications Consensus Group, *IQR* interquartile range, *ICU* intensive care unit

Statistically significant differences are in **bold** type

Regarding specific complications, there was no difference in the occurrence of pneumonia, anastomotic complications, or wound dehiscence. The occurrence of pneumonia was 11.7% in the neoadjuvant group and 11.3% in the upfront-surgery group. Likewise, there was no significant difference in occurrence of either anastomotic complications (3.4 and 4.7%) or wound dehiscence (2.1 and 1.8%) between the neoadjuvant and upfront-surgery groups.

The most common complication 90 days after surgery regarding the ECCG upper-level categories were infectious complications in the neoadjuvant group and gastrointestinal complications in the upfront-surgery group. The most common complications in the neoadjuvant group versus the upfront-surgery group were infectious (20.3 vs. 16.7%), pulmonary (17.6 vs. 14.3%), and gastrointestinal (15.2 vs. 19.6%) complications. None of these differences were statistically significant in any of the analyses. However, cardiac complications occurred significantly more commonly in the upfront-surgery group in the crude analysis (3.4 vs. 7.2%), but after adjustment of confounding variables, the association was attenuated (Table 2).

The length of hospital and ICU stays did not differ between the two groups (Table 2). Also, the 90-day reoperations did not differ between the two groups in any of the analyses. The 90-day mortality was 3.4% in the neoadjuvant group and 6.4% in the upfront-surgery group, with a significant difference in crude analysis (OR, 0.46; 95% CI 0.23–0.93). However, this association was attenuated after adjustment of confounding variables (Table 3).

**Table 3:** Complications after gastrectomy compared between neoadjuvant therapy and upfront surgery^a^

	Main analysis (*n* = 1769)		Sensitivity analysis for R0 resection only (*n* = 1355)	
	Neoadjuvant therapy OR (95% CI)	Upfront surgery OR (95% CI)	Neoadjuvant therapy OR (90% CI)	Upfront surgery OR (95% CI)
Major complications				
Crude	1.122 (0.807–1.562)	1.00 (reference)	1.134 (0.778–1.653)	1.00 (reference)
Model 1^b^	1.104 (0.758–1.607)	1.00 (reference)	1.154 (0.747–1.782)	1.00 (reference)
Model 2^c^	1.120 (0.768–1.632)	1.00 (reference)	1.163 (0.752–1.798)	1.00 (reference)
ECCG 90-day complications				
Pulmonary				
Crude	1.282 (0.917–1.794)	1.00 (reference)	1.152 (0.780–1.700)	1.00 (reference)
Model 1^b^	1.276 (0.867–1.877)	1.00 (reference)	1.146 (0.731–1.796)	1.00 (reference)
Model 2^c^	1.288 (0.874–1.898)	1.00 (reference)	1.156 (0.736–1.815)	1.00 (reference)
Pneumonia				
Crude	1.043 (0.705–1.545)	1.00 (reference)	0.973 (0.617–1.532)	1.00 (reference)
Model 1^b^	1.009 (0.647–1.573)	1.00 (reference)	0.949 (0.565–1.595)	1.00 (reference)
Model 2^c^	1.019 (0.652–1.593)	1.00 (reference)	0.958 (0.569–1.613)	1.00 (reference)
Cardiac				
Crude	**0.458 (0.237–0.887)**	1.00 (reference)	0.559 (0.276–1.131)	1.00 (reference)
Model 1^b^	0.945 (0.453–1.972)	1.00 (reference)	1.272 (0.568–2.848)	1.00 (reference)
Model 2^c^	0.957 (0.458–2.002)	1.00 (reference)	1.269 (0.566–2.845)	1.00 (reference)
Gastrointestinal				
Crude	0.733 (0.519–1.036)	1.00 (reference)	0.730 (0.488–1.093)	1.00 (reference)
Model 1^b^	0.877 (0.595–1.290)	1.00 (reference)	0.843 (0.534–1.329)	1.00 (reference)
Model 2^c^	0.886 (0.601–1.306)	1.00 (reference)	0.847 (0.537–1.338)	1.00 (reference)
Anastomotic complications				
Crude	0.730 (0.371–1.434)	1.00 (reference)	0.826 (0.386–1.772)	1.00 (reference)
Model 1^b^	0.560 (0.268–1.170)	1.00 (reference)	0.531 (0.230–1.227)	1.00 (reference)
Model 2^c^	0.557 (0.266–1.167)	1.00 (reference)	0.530 (0.229–1.226)	1.00 (reference)
Urologic				
Crude	0.753 (0.383–1.481)	1.00 (reference)	0.999 (0.481–2.074)	1.00 (reference)
Model 1^b^	1.104 (0.515–2.366)	1.00 (reference)	1.415 (0.607–3.303)	1.00 (reference)
Model 2^c^	1.109 (0.516–2.384)	1.00 (reference)	1.430 (0.610–3.352)	1.00 (reference)
Thromboembolic				
Crude	1.020 (0.421–2.474)	1.00 (reference)	0.786 (0.231–2.678)	1.00 (reference)
Model 1^b^	1.161 (0.425–3.170)	1.00 (reference)	0.941 (0.236–3.748)	1.00 (reference)
Model 2^c^	1.165 (0.424–3.198)	1.00 (reference)	0.940 (0.236–3.752)	1.00 (reference)
Neurologic				
Crude	0.769 (0.298–1.986)	1.00 (reference)	0.735 (0.255–2.122)	1.00 (reference)
Model 1^b^	1.126 (0.389–3.258)	1.00 (reference)	1.186 (0.361–3.895)	1.00 (reference)
Model 2^c^	1.110 (0.382–3.226)	1.00 (reference)	1.183 (0.359–3.902)	1.00 (reference)
Infectious				
Crude	1.274 (0.928–1.749)	1.00 (reference)	**1.502 (1.047–2.154)**	1.00 (reference)
Model 1^b^	1.215 (0.847–1.744)	1.00 (reference)	1.441 (0.948–2.192)	1.00 (reference)
Model 2^c^	1.239 (0.861–1.783)	1.00 (reference)	1.466 (0.962–2.236)	1.00 (reference)
Wound				
Crude	1.056 (0.435–2.568)	1.00 (reference)	0.594 (0.178–1.985)	1.00 (reference)
Model 1^b^	0.914 (0.337–2.475)	1.00 (reference)	0.565 (0.151–2.019)	1.00 (reference)
Model 2^c^	0.910 (0.335–2.471)	1.00 (reference)	0.563 (0.150–2.104)	1.00 (reference)
Wound dehiscence				
Crude	1.181 (0.482–2.895)	1.00 (reference)	0.647 (0.193–2.173)	1.00 (reference)
Model 1^b^	0.962 (0.350–2.645)	1.00 (reference)	0.555 (0.148–2.083)	1.00 (reference)
Model 2^c^	0.958 (0.348–2.638)	1.00 (reference)	0.554 (0.147–2.082)	1.00 (reference)
Other				
Crude	1.418 (0.642–3.135)	1.00 (reference)	1.973 (0.814–4.780)	1.00 (reference)
Model 1^b^	1.509 (0.600–3.794)	1.00 (reference)	2.030 (0.705–5.847)	1.00 (reference)
Model 2^c^	1.573 (0.622–3.980)	1.00 (reference)	2.135 (0.728–6.257)	1.00 (reference)
90-Day reoperation				
Crude	0.778 (0.465–1.299)	1.00 (reference)	0.740 (0.406–1.351)	1.00 (reference)
Model 1^b^	0.613 (0.348–1.078)	1.00 (reference)	0.641 (0.328–1.251)	1.00 (reference)
Model 2^c^	0.615 (0.349–1.083)	1.00 (reference)	0.644 (0.329–1.260)	1.00 (reference)
90-Day mortality HR (95% CI)				
Crude	**0.461 (0.230–0.925)**	1.00 (reference)	0.397 (0.142–1.112)	1.00 (reference)
Model 1^b^	0.713 (0.331–1.535)	1.00 (reference)	0.688 (0.224–2.114)	1.00 (reference)
Model 2^c^	0.707 (0.327–1.526)	1.00 (reference)	0.693 (0.225–2.134)	1.00 (reference)

^a^Sensitivity analysis included only patients with R0 resection

^b^Model 1: adjusted for age (continuous), sex (male/female), Charlson comorbidity score (0, 1, 2, or ≤ 3), year of surgery (continuous 2005–2016), pathologic cancer stage (0, I, II, III, or IV), surgical technique (open or laparoscopic), and type of gastrectomy (total or other)

^c^Model 2: adjusted for aforementioned confounders, prealbumin (abnormal or normal) and body mass index (BMI) (abnormal or normal)

Statistically significant differences are in **bold** type

The sensitivity analysis with R0 resection alone (*n* = 1355) or with clinical stage instead of pathologic stage suggested results similar to those from the main analysis. Sensitivity analysis with R0 resection suggested only a more common occurrence of infectious complications in the neoadjuvant group (OR, 1.50; 95% CI 1.05–2.15). However, after adjustment of confounding variables, this association was attenuated (Tables 3 and 4).

**Table 4:** Sensitivity analysis of complications compared between neoadjuvant therapy and upfront surgery^a^

	Neoadjuvant therapy (*n* = 1769) OR (95% CI)	Upfront surgery OR (95% CI)
Major complications		
Model 1^b^	1.016 (0.692–1.492)	1.00 (reference)
Model 2^c^	1.041 (0.705–1.537)	1.00 (reference)
ECCG 90-day complications		
Pulmonary		
Model 1^b^	1.138 (0.764–1.695)	1.00 (reference)
Model 2^c^	1.155 (0.771–1.732)	1.00 (reference)
Pneumonia		
Model 1^b^	0.942 (0.597–1.488)	1.00 (reference)
Model 2^c^	0.959 (0.604–1.525)	1.00 (reference)
Cardiac		
Model 1^b^	0.888 (0.419–1.879)	1.00 (reference)
Model 2^c^	0.912 (0.428–1.944)	1.00 (reference)
Gastrointestinal		
Model 1^b^	0.870 (0.585–1.293)	1.00 (reference)
Model 2^c^	0.886 (0.594–1.321)	1.00 (reference)
Anastomotic complications		
Model 1^b^	0.522 (0.245–1.111)	1.00 (reference)
Model 2^c^	0.515 (0.241–1.100)	1.00 (reference)
Urologic		
Model 1^b^	1.021 (0.471–2.211)	1.00 (reference)
Model 2^c^	1.038 (0.475–2.266)	1.00 (reference)
Thromboembolic		
Model 1^b^	1.040 (0.370–2.923)	1.00 (reference)
Model 2^c^	1.058 (0.373–3.007)	1.00 (reference)
Neurologic		
Model 1^b^	1.254 (0.420–3.746)	1.00 (reference)
Model 2^c^	1.237 (0.411–3.724)	1.00 (reference)
Infectious		
Model 1^b^	1.116 (0.771–1.616)	1.00 (reference)
Model 2^c^	1.152 (0.790–1.680)	1.00 (reference)
Wound		
Model 1^b^	1.093 (0.393–3.041)	1.00 (reference)
Model 2^c^	1.090 (0.388–3.062)	1.00 (reference)
Wound dehiscence		
Model 1^b^	1.163 (0.409–3.308)	1.00 (reference)
Model 2^c^	1.151 (0.402–3.296)	1.00 (reference)
Other		
Model 1^b^	1.257 (0.495–3.196)	1.00 (reference)
Model 2^c^	1.339 (0.521–3.440)	1.00 (reference)
90-day reoperation		
Model 1^b^	0.620 (0.348–1.107)	1.00 (reference)
Model 2^c^	0.628 (0.350–1.125)	1.00 (reference)
90-day mortality HR (95% CI)		
Model 1^b^	0.589 (0.273–1.273)	1.00 (reference)
Model 2^c^	0.589 (0.271–1.283)	1.00 (reference)

^a^Adjusted for clinical stage instead of pathologic stage

^b^Model 1: adjusted for age (continuous), sex (male/female), Charlson comorbidity score (0, 1, 2, or 3 ≤), year of surgery (continuous 2005–2016), pathologic cancer stage (0, I, II, III, or IV), surgical technique (open or laparoscopic), and type of gastrectomy (total or other)

^c^Model 2: adjusted for aforementioned confounders, prealbumin (abnormal or normal), and BMI (abnormal or normal)

*OR* odds ratio, *CI* confidence interval, *HR* hazard ratio

Statistically significant differences are in **bold** type

## Discussion

The current study is the first nationwide population-based study and the largest study comparing postoperative complications after neoadjuvant therapy for clinical stage IB or greater gastric adenocarcinoma compared with upfront surgery. The results suggest no increase in major postoperative complications after neoadjuvant therapy. Furthermore, no increase in pneumonia, anastomotic complications, wound complications, or other complications was observed.

Some previous studies have examined postoperative complications after neoadjuvant therapy compared with upfront surgery. The Michigan Appropriateness Guide to Intravenous Catheters (MAGIC) study in 2006 (*n* = 503)^6^ suggested a similar incidence of postoperative complications between patients receiving neoadjuvant therapy (ECF) and those receiving upfront surgery for gastric cancer. Several smaller retrospective studies have repeated these results.^8,21–24^

A meta-analysis^24^ comparing neoadjuvant chemotherapy followed by surgery with surgery alone for locally advanced gastric cancer (*n* = 3362) suggested no difference in major complications, as graded by the Clavien-Dindo classification, between the group receiving neoadjuvant chemotherapy and the group receiving upfront surgery. Likewise, our study found no difference in the occurrence of major postoperative complications (Clavien-Dindo ≥ III). A Chinese study (*n* = 238) estimated the occurrence of major postoperative complications at 8.8% and the total occurrence of complications at 17.2% for patients receiving neoadjuvant chemotherapy for gastric adenocarcinoma.^21^ The higher incidence of major complications in our study compared with in studies from Eastern countries could be explained by the real-world population-based design with older and more comorbid patients. Taken together, the studies show that neoadjuvant therapy does not seem to increase major postoperative complications in gastric cancer.

The aforementioned meta-analysis^24^ also suggested fewer anastomotic leaks after neoadjuvant therapy than after upfront surgery. However, our study showed no significant association between neoadjuvant therapy and anastomotic leaks. The meta-analysis reported no significant difference in the occurrence of pneumonia or wound infections between patients receiving neoadjuvant therapy and those undergoing surgery alone, in line with the current study.

In 2011, a Chinese study^9^ compared complications after FOLFOX7 neoadjuvant chemotherapy and upfront surgery. They suggested that the most common surgical complications for patients receiving neoadjuvant therapy for gastric cancer were anastomotic leak and intra-abdominal abscess. In the current analysis, these complications were similarly frequent in both the neoadjuvant and upfront-surgery groups. The Chinese study^9^ also suggested that preoperative neoadjuvant chemotherapy led to a statistically significantly longer hospital stay (13 vs. 11 days), whereas our study found no difference in the length of hospital or ICU stay. The meta-analysis^24^ suggested that neoadjuvant chemotherapy would lead to a lower reoperation rate, but the results of the current study suggested no difference in the 90-day reoperation rate. Previous studies have reported no difference in short-term mortality between neoadjuvant therapy and upfront surgery for gastric cancer,^6,9,24^ in line with the results of the current study.

The main strength of this study was its population-based nationwide design, reducing selection bias. The large size of the cohort was another strength. Compared with previous studies, the current study comprehensively assessed and categorized complications, which increases its comparability with other studies. The most significant confounding factors were taken into account in the analysis, including age, Charlson Comorbidity Index, year of surgery, pathologic cancer stage, surgical technique, type of gastrectomy, albumin, and BMI.

However, the current study also had weaknesses. Because it was a retrospective study, there was always the possibility that some complications may have been missed during review of patient records. However, the incidence of complications was similar to that of a Dutch prospective study using the same definitions, suggesting that the complications were identified correctly.^5^ Also, the proportion of patients receiving neoadjuvant chemotherapy was low but increased over time. Furthermore, the majority of the patients received EOX chemotherapy and derivatives, which have to some extent been replaced by FLOT for fit patients in recent years,^25^ and the results may not necessarily be applicable to patients receiving FLOT. However, EOX and derivatives still are used as first-line treatment in many centers around the globe, as well as for patients not fit for FLOT therapy. As expected, the patients in the neoadjuvant group were younger and healthier than those in the upfront-surgery group, but this was taken into account in the analysis.

Finally, it could be argued that the number of patients undergoing laparoscopic gastrectomy was low. However, the analysis adjusted for the type of surgery (laparoscopic or open), and neoadjuvant treatment should not greatly modify the effects of type of surgery on complications. Furthermore, a Chinese study in 2022 found no significant difference in the occurrence of Clavien-Dindo grade II or greater complications between open and laparoscopic gastrectomy for patients receiving neoadjuvant therapy for gastric cancer.^26^

The current study is the largest and first population-based nationwide study on this topic. Based on the data, neoadjuvant therapy can be safely administered to patients with gastric cancer without increasing the risk of postoperative problems. The results can inform oncologists, surgeons, and clinical treatment guidelines on the potential effects of neoadjuvant treatment on surgical risk for gastric cancer patients.

In conclusion, this population-based nationwide study suggests no increase in postoperative complications, reoperations, or short-term mortality after neoadjuvant therapy compared with upfront surgery for gastric adenocarcinoma.

## Data Availability

The data can be shared for research purposes upon request by contacting the Chief Investigator, Professor Joonas Kauppila, but may be restricted by and require complimentary permissions from the ethical committee and relevant original data holders.
